# Review of the use of Topiramate for treatment of psychiatric disorders

**DOI:** 10.1186/1744-859X-4-5

**Published:** 2005-02-16

**Authors:** Danilo Arnone

**Affiliations:** 1 Department of Psychiatry, Springfield University Hospital, St George's Medical School, London, UK

**Keywords:** topiramate, mood stabilisers, psychotropic medications, psychiatric disorders.

## Abstract

**Background:**

Topiramate is a new antiepileptic drug, originally designed as an oral hypoglycaemic subsequently approved as anticonvulsant. It has increasingly been used in the treatment of numerous psychiatric conditions and it has also been associated with weight loss potentially relevant in reversing weight gain induced by psychotropic medications. This article reviews pharmacokinetic and pharmacodynamic profile of topiramate, its biological putative role in treating psychiatric disorders and its relevance in clinical practice.

**Methods:**

A comprehensive search from a range of databases was conducted and papers addressing the topic were selected.

**Results:**

Thirty-two published reports met criteria for inclusion, 4 controlled and 28 uncontrolled studies. Five unpublished controlled studies were also identified in the treatment of acute mania.

**Conclusions:**

Topiramate lacks efficacy in the treatment of acute mania. Increasing evidence, based on controlled studies, supports the use of topiramate in binge eating disorders, bulimia nervosa, alcohol dependence and possibly in bipolar disorders in depressive phase. In the treatment of rapid cycling bipolar disorders, as adjunctive treatment in refractory bipolar disorder in adults and children, schizophrenia, posttraumatic stress disorder, unipolar depression, emotionally unstable personality disorder and Gilles de la Tourette's syndrome the evidence is entirely based on open label studies, case reports and case series. Regarding weight loss, findings are encouraging and have potential implications in reversing increased body weight, normalisation of glycemic control and blood pressure. Topiramate was generally well tolerated and serious adverse events were rare.

## Background

The use of mood stabilizing antiepileptic drugs has increasingly been explored for the treatment of different psychiatric conditions. Topiramate is a novel neurotherapeutic agent approved in more than 75 countries for adjunctive treatment for refractory partial-onset seizures or primary generalised tonic-clonic seizure in adults and children over 2 years of age and migraine prophylaxis in USA. Several mechanisms of action of topiramate support the hypothesis for its putative actions in bipolar affective disorders, unipolar depression, schizophrenia, posttraumatic stress disorder, disordered eating behaviour. This article reviews the pharmacology of topiramate and describes adverse events and the outcomes observed in published and unpublished studies. Particular interest is focused on topiramate related weight loss and its clinical implications.

### Pharmacokinetic and pharmacodynamic profile

Topiramate is a sulfamate substituted, derivative of the monosaccharide D-fructose [1]. It is absorbed in 1–4 hours, its oral bioavailability is about 80% and its plasma protein binding is 15%. It pharmacokinetic profile is linear in relation to dose [2]. It does not affect liver enzymes, it is excreted unchanged in the urine, and has a high therapeutic index [3]. In renal impairment, the clearance of topiramate is decreased and elimination half-life is prolonged, usually between 19 and 23 hours [4]. Moderate, not clinically significant, increases in plasma concentrations have been observed in the presence of hepatic disease [2,4]. It is not extensively metabolised, and six inactive metabolites have been identified [4]. Topiramate half-life (18–23 hrs) is decreased by carbamazepine [5]. It may compromise the efficacy of oral contraceptive agents by reducing mean total exposure to the estrogen component [6]. Similarly to carbamazapine and valproate, topiramate reduces the seizure threshold and the after-charge duration in the amygdale-kindled rat [7]. It may increase cerebral GABA concentrations in humans [8], enhancing the inhibitory GABAergic transmission by binding to allosteric GABA-A receptors, probably through a non-benzodiazepine mechanism and second-messenger systems [9,10]. Also, topiramate may inhibit brain glutamate release, by antagonising α-amino-3-hydroxy-5-methyl-4-isoxazolapropionate (AMPA) kainate type of glutamate receptors, and may inhibits NA (+) and L-type Ca (2+) channel neuronal activities [11,12]. Topiramate is also suggested to be an inhibitor of specific carbonic anhydrase isoenzymes [13].

### Rationale for evaluating topiramate in psychiatric disorders

The use of topiramate in bipolar spectrum disorders is based on the putative shared biological mechanism between epilepsy and bipolar disorders suggested by the amygdala-kindled seizures in animal models [14-16] and the high rate of co-morbid psychiatric conditions in epilepsy [17]. However, there is inadequacy of current treatment strategies [18]. The efficacy of lithium, valproate, and carbamazapine in prophylaxis of bipolar spectrum disorders is rather modest [19-22]. Mixed or rapid cycling disorders are particularly characterised by a poor response to lithium treatment, which reaches 72–82% [23,24]. Twenty-five to fifty percent of patients need reduction or discontinuation of lithium therapy due to adverse effects [25] and up to 55 % of patients develop resistance to lithium after 3 years of treatment [26]. Pharmacological interventions are also limited in bipolar depression extensively treated with antidepressants [27] in the absence of replicated controlled studies [21], and with the recognised risk of induced hypomanic switching or cycles acceleration [28-30]. Lamotrigine has demonstrated stabilising properties in bipolar I depression and rapid cycling bipolar II disorder [31,32] highlighting the role of newer mood stabilisers in the treatment of this condition. In unipolar major depression the role of double-blind placebo controlled trials confirm that lithium is effective in about 40–50% of patients and there is scope for the use of mood stabilising agents such as carbamazapine and sodium valproate [33]. In schizophrenia, the postulated action of anticonvulsants is based on some evidence supporting a reciprocal interaction between glutamatergic and dopaminergic systems. It is postulated that the striatum, which has rich D1 and D2 dopamine innervation, receives cortical, limbic and thalamic excitatory glutamatergic afferents. Striatal activation by glutamate leads to inhibition of the thalamic sensory outflow to the cortex. This effect seems to be mediated by inhibitory gabaergic neurons acting via thalamic circuits [34]. Phencyclidine binds non-competitively to a site adjacent to N-methyl-D-aspartate (NMDA) receptor of glutamate exercising an inhibitory effect that can mimicry schizophrenia. This model constitutes the theory for the 'hypothesis of glutamatergic hypofunction' based on receptor hypofunction or 'glutamatergic deficiency' in the pathophysiology of schizophrenia [35-37]. In humans with schizophrenia, elevated levels of N-acetyl-aspartyl-glutamate, a naturally occurring acidic dipeptide, could dampen or antagonize NMDA receptor mediated neurotransmission. Elevated levels of N-acetyl-aspartyl-glutamate could rise from diminished activity of glutamate carboxypeptidase II, a hydrolytic enzyme enriched at glutamatergic nerve terminals and located on the membrane of astrocytes [35,38]. Topiramate mechanisms of action could optimise the imbalanced availability of glutamate and/or GABA in the subcortical circuitation.

Posttraumatic stress disorder can be a difficult condition to treat especially if the course is chronic [39]. Current pharmacological interventions are limited to serotoninergic reuptake inhibitors (SSRIs). Hypothesis on the aetiology of posttraumatic stress disorder, have suggested that after exposure to traumatic events, limbic nuclei may become kindled and sensitized. Consequently, drugs known to have anti-kindling or anticonvulsant effects might have potential in the treatment of posttraumatic stress disorder [40]. Carbamazapine and valproate may be effective [41,42]. Particularly carbamazapine has shown efficacy in reducing re-experiencing and arousal symptoms whilst valproate decreased avoidance/numbing and arousal symptoms [43]. Most recently lamotrigine has also shown some efficacy [44]. The use of topiramate in eating disorders derives from the observation of appetite suppression and weight loss in controlled trials in patients with epilepsy [45]. Animal models suggest that stimulation of the lateral hypothalamus, by glutamate agonists (like kainate/AMPA agonists), causes an intense rapid dose-dependent increase in food intake [46,47]. Antagonists of kainate/AMPA glutamate receptors like topiramate might contribute to suppress appetite and to regain control over eating, a typical feature observed in eating disorders [48]. Clinically, this would be in agreement with EEG abnormalities found in bulimia nervosa. Another postulated mechanism might be linked to a recent observation that topiramate down-regulates neuropeptide Y1 and 5 receptor subtypes in rats [49]. Current pharmacological approaches to treatment of binge eating disorders are limited to SSRIs [50] and imipramine [51] whereas desipramine [52] and d-fenfluramine [53] have not been associated with weight loss. Similarly, in bulimia nervosa, SSRIs constitute the main pharmacological resource [54], with some possible effectiveness for carbamazapine [55] and phenytoin [50,56,57].

In alcohol dependence, antiepileptic medications share neurochemical effects with alcohol by inhibiting neuronal excitation. Carbamazapine, gabapentin, and valproic acid have been reported to reduce alcohol consumption [58]. Chronic alcohol intake is linked to decreased GABA receptor activity in the ventral tegmental area with disinhibition of dopaminergic neurons [59]. Similarly, hippocampal and cortical GABA neurons projecting to the midbrain might facilitate dopaminergic neurotransmission in the midbrain at glutamate binding sites [60] such as kainate/AMPA receptors [61]. The putative efficacy of topiramate in the treatment of alcohol dependence is based on reversing chronic changes induced by alcohol resulting in dopamine-facilitated neurotransmission in the midbrain. In psychiatry, drug induced severe obesity plays an important role [62] and substantive weight gain has been described with several psychotropic medications [63-65]. Obesity is associated with an increase risk of co-morbid medical conditions such as hypertension, diabetes and cardiovascular disease [66]. Diabetes mellitus reaches nearly 10% prevalence among hospitalized subjects with bipolar disorder in USA [67]. Topiramate induced weigh loss in the 5–10% range is associated with significant reduction in blood pressure and changes in total cholesterol, low-density lipoproteins and triglycerides [68]. There are no clear mechanisms underlying weight changes but it may be dependent of glycemic control as suggested by Chengappa et al. [69].

## Methods

A comprehensive search from a range of electronic databases, including BNI, CancerLit, Cochrane Library, EMBASE, Medline, Psychinfo, and Pub MED was conducted for the period from the introduction of topiramate to December 2003. Key words used to identify the studies were: TOPIRAMATE or ANTICONVULSANTS and PSYCHIATRIC DISORDERS, PSYCHIATRY, PSYCHOSIS, AFFECTIVE DISORDERS, EATING DISORDERS, SCHIZOPHRENIA, SCHIZOAFFECTIVE DISORDERS. The search was also complemented by manual search of bibliographic cross-referencing. Researchers who had expressed an interest in the subject were contacted for any non-published information. Janssen-Cilag Ltd medical information was also contacted. There was no restriction on the identification of studies in terms of publication status, language and design type. Papers were identified if presented original data and addressed the question, 'use of topiramate in treating psychiatric conditions'. Studies were screened for design type, diagnosis according to diagnostic criteria, topiramate dose, titration regime, response onset, response rate, duration of treatment, outcome measures, and adverse events. Presence of weight loss (preferably expressed as ≥5% reduction in baseline weight) was also considered. Response was preferably indicated by significant score reduction in rating scales or objective measures. Randomised controlled studies if available were considered primary source of evidence, followed by naturalistic studies, case series and case reports. Reports or posters presented to meetings and subsequently re-considered in larger numbers or published were excluded.

## Results

Thirty-two published reports met criteria for inclusion, 4 controlled and 28 uncontrolled studies (see Additional file-table 3). Five unpublished controlled studies were identified in the treatment of acute mania (table 2). Details are given below.

**Table 2:** Characteristics of the studies included in the review

**Condition**	**Number of studies**	**Design**	**Outcome**
**Bipolar disorders**			
			
*Bipolar mania*	8	5 controlled (*)	Negative
		3 open label (70–72)	Positive
			
*Rapid-cycling bipolar disorders*	1	Open label, add-on (75)	Positive
			
*Adjunctive therapy (refractory bipolar disorders)*	12	Open label (76–87)	Positive
			
*Bipolar depression*	2	1 Controlled, add-on (88)	Positive
		1 Open label, add-on (89)	Positive
			
*Bipolar disorders in children and adolescents as adjunctive treatment*	1	Open label, add-on (90)	Positive
			
**Unipolar depression**	2	1 Case report (91)	Negative
		1 Chart review (92)	Positive
			
**Schizophrenia, schizoaffective disorders and psychosis unspecified**	3	2 Case series (93, 94)	Negative
		1 Case report (95)	Positive
			
**Eating disorders and disordered eating**	4	2 Controlled (96, 97)	Positive
		1 Open label, add on (98)	Positive
		1 Case series, add on (99)	Positive
**Posttraumatic stress disorder**	1	Open label, add on (100)	Positive
			
**Alcohol dependence**	1	Controlled (101)	Positive
			
**Gilles de la Tourette's syndrome**	1	Case series (102)	Positive
			
**Emotional unstable personality disorder**	1	Case reports (103)	Positive

(*) Unpublished

### Bipolar disorders

#### Bipolar mania

Following encouraging results from preliminary reports in acute mania [70-72], topiramate was compared with placebo in one double-blind randomised trial [73]. Two different dosages of topiramate (250 and 500 mg/day) were studied in a 3-week trial among hospitalised patients. The final analysis found no significant differences in efficacy in the three groups. Four subsequent large unpublished placebo controlled studies, unavailable for review, failed to demonstrate efficacy of topiramate in mania compared to placebo, leading to the discontinuation of development programs [[74]; Calabrese, personal communication].

#### Rapid-cycling bipolar disorders

*Kusumakar et al. *[75] studied 27 women with ultra rapid, ultradian, and chaotic biphasic bipolar disorder type I/II refractory to treatment for 16 weeks and more than 29% weight gain over the previous 24 months. The study had a prospective open label, add-on design. Topiramate was introduced at a dose of 25 mg/day, and increased by 25 mg/day every 5–7 days until clinical response or tolerability was reached. The dose range was 100–150 mg/day. Rating scales used in this study were the Hamilton depression rating scale, 21 items (HAM-D-21), the Young mania rating scale (YMRS), and daily assessments of mood, sleep pattern, and weight loss. Among the 23 patients who completed the study, clinical response was noted within 12 weeks for 15 patients who remained euthymic for at least 4 weeks. Weight loss >5% was recorded in 9 patients and of 1–4% in 5 patients. The rest of the subjects experienced no weight change and in 1 case weight gain was recorded. Only 4 patients discontinued the study because of adverse events (drowsiness and dizziness, ataxia, confusion, inability to concentrate).

### Adjunctive therapy in treatment-refractory bipolar disorders

*Marcotte et al. *[76] in an open-label study examined retrospectively 58 in-out patients with different psychiatric disorders, refractory to conventional mood stabilisers, and with psychiatric and medical co-morbid conditions. Forty-four patients had rapid cycling bipolar disorder (manic, hypomanic and mixed), 9 had schizoaffective disorder, 3 had dementia, and 2 had psychotic illness. The range of duration of psychiatric illness was from 7 months to 40 years. The mean duration of topiramate treatment was 16.0 weeks with a mean dosage of 200 mg/day (range 25–400 mg/day). The initial dose was 25 mg twice daily, slowly increased by 50 mg every 7 days. Response was regarded as 'marked' or 'moderate' improvement based on a Likert global assessment scale including quality of sleep, appetite, mood, and concentration during therapy. Twenty-three (52%) of the 44 rapid cycling bipolar disorder patients and 36 (62%) of the whole sample showed 'marked' or 'moderate'. Six (46%) of the 13 patients with rapid cycling bipolar disorder and substance misuse showed marked or moderate improvement when topiramate was added. Adverse effects were minor and 6 (10%) patients discontinued due to adverse events (delirium, grand mal seizures, increased panic attacks, confusion, frequent bowel movements, nausea, somnolence, fatigue, impaired concentration and memory, paraesthesias). In a larger cohort continuation of open treatment with topiramate showed additional clinical improvement with longer drug exposure [77].

*Chengappa et al. *[78] examined prospectively in a 5-week naturalistic study 18 patients with a diagnosis of bipolar disorder type I (manic, hypomanic, mixed phase and rapid cycling) and 2 patients with schizoaffective disorder (bipolar type), all refractory to previous mood stabilizing therapies. Topiramate was added on to existing pharmacotherapy and it was initiated at a dosage of 25 mg/day, increased by 25–50 mg/day every 3–7 days. The target dose was in the 100–300-mg/day range. The YMRS, HAM-D-21, and the clinical global impression scale for bipolar disorder (CGI-BD) were used in the evaluation. Response was defined as 50% or greater reduction in the total Y-MRS scores and CGI-BD score of 'much or very much improved'. Twelve of the patients (60%) responded to topiramate, within 2–4 weeks after treatment initiation. Progressive decline in weight and body mass index (BMI) occurred during the course of therapy. Topiramate was well tolerated and adverse events were minor. The average weight loss was 1.5–2 lb/week. Subjects with BMI of 30 or more (i.e. obese) lost more weight.

*McElroy et al. *[79] studied 56 outpatients participating in the Stanley Foundation Bipolar Outcome Network in a prospective study with an open label add-on design. Patients had bipolar disorder type I/II, psychotic disorder not otherwise specified and schizoaffective disorder bipolar type, inadequately responsive or poorly tolerant to one or more standard mood stabilizers. The YMRS, CGI-BD and the Inventory of Depressive Symptoms (IDS) were used in the assessments. The baseline YMRS reflected only mild mania. The initial dose was 25–50 mg/day, given either at night or in divided doses, subsequently increased every 3–14 days by 25–50 mg/day, according to patients' response and side effects. The maximum dose utilised was 1200 mg/day. The mean dose at 10 weeks was 193.2 mg/day (SD = 122.0) and 244.7 mg/day (SD = 241.7) at last evaluation. Thirty manic and 11 depressed patients completed the 10 weeks acute phase, of which 19 manic (63.3%) and 3 depressed (27.3%) were 'much or very much improved' so regarded as responders, according to YMRS, CGI-BP-Mania and IDS but not CGI-BP-Depression. Thirty-seven patients continued open maintenance treatment with topiramate for a mean ± SD of 294.6 ± 145.3 days (i.e., more then 7 months): 22 manic, 5 depressed and 10 euthymic patients. At last evaluation, 12 manic patients (55%) were rated as much or very much improved and 10 minimally or no changed, 1 depressed patient was rated very much improved and 4 displayed no or minimal change, 9 euthymic displayed minimal or no change and 1 had worsened with mixed symptoms. In total 29 (52%) discontinued topiramate during the acute and maintenance phase (up to a year). The main reasons for discontinuation were increased depressed (N = 7) or hypomanic/manic (N = 4) symptoms, discontinuation of medication (N = 1) and side effects (N = 6). Ten patients (18%) discontinued topiramate because of side effects. Topiramate was associated with reduction in BMI and body weight. Patients who began topiramate for depressive symptoms or relative euthymia did not display notable changes in ratings at most time points.

*Sacks et al. *[80] treated 14 patients with treatment resistant bipolar disorder and a variety of co-morbid conditions for a mean of 22.4 +/- 22.0 weeks with adjunctive topiramate in a retrospective trial. The mean dose of topiramate was 50 mg/day (SD = 27.4). Among the 11 patients who remained on treatment for longer than 2 weeks, 4 experienced decreased severity of bipolar illness by more than 1 CGI score and 8 experienced significant improvement in their primary co-morbid condition. Four patients with BMI of 28 or more experienced a mean weight loss of 13.5 +/- 7.4 kg whilst on topiramate. Discontinuation occurred in 5 patients due to adverse effects (paraesthesias, rash, cognitive impairment, sedation) and in 2 due to lack of efficacy.

*Eads et al. *[81] studied 17 treatment resistant patients with bipolar disorder type I (N = 11) and II (N = 3). The study was retrospective in design and with a mean duration of 22.4 (SD = 22.0) weeks. Patients were evaluated with the Global Assessment of Functioning (GAF) scores. Topiramate was added to other medications and titrated to a mean dose of 826 mg/day in divided doses. Nine patients completed the study and 8 patients discontinued due to adverse effects (cognitive impairment, sedation, paraesthesias). All nine patients responded to topiramate with 8–20 improvement on the GAF scale. Eight experienced clinically significant improvement in their primary co-morbid condition as measured by the Clinical global impression scale for improvement (CGI-I) (anorexia nervosa N = 1, bulimia N = 3, obesity N = 1, obsessive compulsive disorder N = 1, Tourette's N = 1). Patients with BMI of 28 or more (N = 4) experienced a weight loss of 29.75 lb (SD = 16.29).

*Ghaemi et al. *[82] in a retrospective open label study reviewed 76 charts of outpatients with refractory bipolar disorder type I/ II or psychotic disorder non otherwise specified (depressive phase N = 33, rapid cycling N = 24, mixed episodes N = 8 and prophylaxis N = 8, hypomania N = 3). In all the patients topiramate had been added on or used in monotherapy. The main dose of topiramate used was 96.1 mg/day (SD = 94.19) (range 12.5–400 mg/day) for a mean duration of 17.5 (SD = 16.7) weeks (range 0.5–65 weeks). Response was measured with the CGI-I rating scale as 'moderate' to 'marked' improvement. The overall response rate to topiramate was 13.2% (10/76). Response rates remained similar when assessed on indication of treatment. Responders received a higher dose of topiramate (180 mg/day, SD = 120.1) than non-responders (83.2 mg/day, SD = 83.7, p = 0.002) and higher in the high rather than the low dose group (p = 0.04, Fischer's exact test). Topiramate was not higher in patients receiving monotherapy (N = 6). Response rate between subjects receiving mood stabilisers (p = 0.27 Fischer's exact test) or antidepressant (p = 0.48 Fischer's exact test) and those who did not wasn't significant. Weight estimates were based on patient self-report. Weight loss was experienced by 51.6% of the sample with 14.2 lb (SD = 6.2) (range 5–25 lbs). Topiramate dose was also higher in those subjects who lost weight (138.3 mg/day) than in those who did not (70 mg/day, p = 0.007) but not the amount of weight (p = 0.49). There was no difference if concomitant medication were used (p = 0.43). Side effects were reported by 81.6% of the sample. Topiramate was discontinued in 51.3% (N = 39) of the sample with 27 (69.2%) for side effects (paraesthesias, nausea, fatigue, insomnia, slowed thinking, sedation, ataxia, headache, agitation, frequent peristalsis) and 7 for lack of efficacy.

*Vieta et al. *[83] designed a prospective, 6-week open label study with an add-on design. The authors studied 21 patients with poor response or intolerance to mood stabilisers and with a diagnosis of bipolar disorder type I/II in a manic (N = 9), mixed (N = 2), hypomanic (N = 3) and depressed (N = 6) phase or schizoaffective manic (N = 1). The YMRS, HAM-D-17 and CGI rating scales were used. At study entry, patients had a minimum score of 12 on YMRS and HAM-D and a minimum score of 4 on CGI. Topiramate was introduced at the dose of 25 mg/day and increased by 25–50 mg every 3–7 days to a mean dose of 158 mg/day. At end point, among the 15 patients who completed the study, 6 (28.5% by intention to treat) were responders with 50% or greater decrease in YMRS or in HDRS-D-17 scores and 2 or more in the CGI-BP. Patients in the depressed phase only obtained a reduction equal to 50% in HDRS-17. Six patients discontinued for lack of efficacy and side effects (paraesthesias, impaired concentration, anxiety) (N = 1), poor compliance (N = 1) and loss of follow up (N = 3). Ten patients experienced moderate weight loss.

*Saxena et al. *[84] assessed the efficacy of topiramate as adjunctive treatment in 9 bipolar disorder patients resistant to conventional mood stabilisers, in a prospective 10–24 week open label trial. Significant decrease in YMRS and HAM-D were observed in four patients. Decreases in CGI-I in the Global assessment scale (GCI-S) scores of at least one point from baseline to endpoint were noted in all patients and no relapses were observed. Topiramate was titrated according to efficacy with a mean dose at endpoint of 488 mg/day. It was well tolerated at doses of up to 600 mg/day. The mean weight loss during the follow up period was 5.39 kg. Only one patient discontinued due to side effect (anxiety, sleep disturbance, lack of libido).

*Vieta, Torrent et al. *[85] completed a 6-month open trial with 34 treatment resistant bipolar patients (type I = 28, type II = 3, not otherwise specified = 2 and schizoaffective = 1) in different phases (manic = 17, depressive = 11, hypomanic = 3, mixed = 3). Topiramate therapy was added on current medication and the dose titrated slowly. The dose at end point was 202 mg/day (SD = 65). Outcome measures included the YMRS, HAM-D, and CGI for severity. Twenty-five patients (74%) completed the study, 9 subjects discontinued due to lost of follow up (N = 4), worsening of symptoms (N = 2), side effects (N = 1), hospitalization (N = 1) and non-compliance (N = 1). Response occurred within 2–6 weeks. Fifty-nine percent of manic patients and 55% of depressed patients responded to the drug by intention to treat analysis expressed as significant reduction in rating scales. Only one patient discontinued due to side effects (paraesthesias) and topiramate was generally well tolerated.

*Vieta, Ros, Valle et al. *[86] evaluated 61 refractory bipolar patients, in a 12-week preliminary multicentre study. Outcome measures included the YMRS, HDRS and CGI-BP. The mean YMRS at baseline was 27.8. Among the 55 patients who completed the study, 43 patients (70%) were considered responders with 50% or more reduction in YMRS score. Also 25 patients (41%) met criteria for remission with YMRS score of 8 or less. Weight loss was recorded in 24 (39%) patients. Those with the highest BMI at baseline (>40) experienced the greatest weight loss (mean 3.3 kg) during the follow up. Highly significant reduction in HDRS (p = 0.004) and CGI-BP (p < 0.0001) from baseline to endpoint were also noted. Only 6 patients discontinued the study due to loss of follow up (N = 2), non-compliance (N = 2), lack of efficacy (N = 1), and side effects (paraesthesias) (N = 1). The mean topiramate dose at endpoint was 214 mg/day.

*McIntyre et al. *[87] enrolled 109 subjects with bipolar disorder type I/II in manic (N = 3), hypomanic (N = 18), mixed (N = 33), depressed (N = 40), rapid cycling (N = 15) phases, resistant to conventional antipsychotics in a 16-week, add-on, naturalistic trial. Different co-morbid disorders were present in 24 subjects. The baseline YMRS score was 13 or greater, the Montgomery and Asberg depression rating scale (MADRAS) was 12 or greater, and the CGI-S was 'moderate', 'marked' or 'extremely severe'. Topiramate mean dose was 140.8 mg/day (range 25–400 mg/day). Seventy patients completed the study but 99 were evaluable at end point. Seventy percent of subjects (N = 69) responded to topiramate treatment with a reduction of 50% or more on YMRS score. Twenty-five subjects obtained remission at endpoint expressed as YMRS of 8 or less. The MADRAS score decreased in the all population studied throughout the study period, with a more pronounced decrease in subjects not on antidepressants (N = 57). Sixty percent (N = 59) of patients responded to topiramate according to MADRAS expressed as 50% or more reduction in score and 37 obtained remission defined as a score of 12 or less. Thirty-nine subjects discontinued because of adverse events (paraesthesias, nausea, fatigue, somnolence, frequent peristalsis, blurred vision, headache, dizziness) (N = 12), lack of efficacy (N = 6), missed doses (N = 3), protocol violation (N = 5), withdrew consent (N = 9), lost at follow up (N = 3), other reasons (N = 1). Adverse events occurred in 131 patients. Tremor, scored with the VAS severity scale (1–10 range), showed a reduction in severity from 3.84 at baseline to 2.06 at week 16 (p < 0.001). Subjects' satisfaction with treatment was also considered with only 10% of patients rated 'completely dissatisfied', 'somewhat dissatisfied', 'neither satisfied nor dissatisfied'. Weight change was noted in 107 subjects: 65 lost weight, 24 gained weight and 18 maintained their weight. It was not evaluable in 2 patients. The mean weight change at endpoint was – 1.8 Kg (p < 0.001).

### Bipolar depression

*McIntyre et al. *[88] conducted a study where topiramate was added to current medication and randomly compared to bupropion in the treatment of 36 subjects for bipolar disorder type I/II in depressive phase. This was an 8 weeks single blind (rater blinded) study developed in outpatients setting, with intent to treat analysis. Topiramate was introduced at the dose of 50 mg/day and titrated every two weeks until clinical response was obtained to a maximum of 300 mg/day. The mean dose of topiramate was 176 mg/day (SD = 102 mg/day). Fifty-six percent of patients on topiramate and 59% for bupropion obtained 50% or more decrease from baseline in HDRS-17 scores. Response to treatment ranged from two to four weeks. Significant reduction in YMRS and CGI-I scores were also observed at week-8 similarly in both the topiramate and the bupropion SR groups with no significant difference between the two. Weigh loss was recorded in both treatment groups; the mean weight loss was of 1.2 Kg in the bupropion SR group and 5.8 Kg in the topiramate group. Adverse events were reported in eleven (61%) patients receiving topiramate and nine (50%) receiving bupropion SR. In total 8 of patients receiving topiramate and 5 of patients in the bupropion SR group discontinued prematurely. Six patients in the topiramate group and 4 patients in the bupropion SR group discontinued for adverse events (topiramate group: paraesthesias, nausea, sweating, decreased/increased appetite, anxiety, slow memory, word finding difficulty, tremor, blurred vision and headache). The two further discontinuations in the topiramate group were attributable to lack of efficacy (N = 1) and withdraw of consent (N = 1).

*Hussein et al. *[89] studied the efficacy of topiramate as adjunctive treatment with a 3-year, naturalistic study in patients with bipolar disorder type I (N = 65) and II (N = 18) in a moderately severe depressive phase, refractory to mood stabilisers. Depressive symptomatology was assessed with the HAM-D-17 scale. Topiramate was commenced at a dose of 50 mg/day and titrated every 2 days to a mean dose of 275 mg/day (range 100–400 mg/day). Forty-one patients completed the study but 65 were evaluable with 35 (54%) who showed great improvement (HAM-D score at endpoint 0–5) and 6 (9%) partially responded (HAM-D score 6–10). The response occurred within the first 4 weeks of treatment. Nineteen patients (29%) abandoned the study because adverse events (paraesthesias, nausea, dizziness). The average weight loss in 36 months was 38 pounds.

### Bipolar disorders in children and adolescents as adjunctive treatment

*DelBello and associates *[90] evaluated topiramate as open label, adjunctive treatment for children and adolescents with bipolar disorder type I/II for 4.1 months (SD = 6.1). The charts of 26 subjects were retrospectively reviewed using the CGI and CGA scales separately for mania and overall bipolar illness. The dose at end point was 104 mg/day (SD = 77). Response rate defined as improvement of 2 or more points on the rating scales was 73% for mania and 62% for overall bipolar disorder. No serious adverse events were reported.

### Unipolar depression

*Gordon and Price *[91] reported topiramate lack of efficacy in a case report of recurrent major depression. Topiramate was used as adjunctive treatment for 8 weeks at a dose of 300 mg/day. Anxiety and depressive features supervened leading to discontinuation. A significant weight loss of 15 lb occurred. *Carpenter and associates *[92] reviewed the charts of 16 females patients with treatment resistant unipolar depression and obesity (mild to moderate) treated with open label adjunctive topiramate. Self reported symptoms and clinician ratings were assessed regularly. Only 36% of patients were considered responders at 5.5 weeks (SD = 1.2) and 44% at end point 17.7 weeks (SD = 13.4). The initial dose of topiramate was 25–100 mg daily, increased variably according to the individual's symptomatology and side effects; the final dose was 277 ± 101 mg/day (range 100–400 mg/day). Four subjects discontinued due to adverse events (paraesthesias, memory concerns, lack of concentration, dysgeusia). Body mass index decreased significantly with a mean weight loss of 6.1 % (SD = 8.2).

### Schizophrenia, schizoaffective disorders and psychosis unspecified

*Millson et al. *[93] in a case series treated 3 men and 2 women with chronic schizophrenia adding topiramate to current medication. The initial dose was 50 mg/day and titrated at 50 mg/week to a mean dose of 250 mg/day (range 200–300 mg/day). Current medication dose was held constant. Positive and negative symptoms were monitored with the Positive and Negative Syndrome Scale for schizophrenia before commencing topiramate and a month after the maximum dose was administered. A deterioration of both positive and negative symptoms was noted in all the subjects.

*Dursun and Deakin *[94] augmented antipsychotic medication with either topiramate or lamotrigine in 26 outpatients with treatment resistant schizophrenia. The case series had an open label, add-on design with 24-week duration. Psychopathology was assessed periodically with the Brief Psychiatric Rating Scale (BPRS) and the baseline score was of at least 30. Nine patients received topiramate in addition to their current treatment and did not show significant reduction at end point compared to the baseline score. Topiramate was initiated at a dose of 25 mg/day and increased to a maximum of 300 mg/day with a range of 225–300 mg/day at end point. Tolerability and side effects were not assessed systematically but no clinically significant or serious side effects were reported. Weight change was not assessed.

*Drapalski et al. *[95] suggested an improvement in negative symptoms in a patient with schizophrenia when added to a stable regimen of antipsychotic medication. The patient described was a participant in a 17 weeks duration open label study with an on-off design. An initial 4-week titration phase was followed by 8-week maintenance phase, 1-week tapering phase and 4-week follow-up. Negative symptoms were assessed with the Negative Scale of the Positive and Negative Syndrome Scale (PANSS) at baseline (Negative Scale score = 24), 4-week, 8-week and follow-up after discontinuation of topiramate. There was a significant 7 points improvement at the end of medication phase (from 24 to 17). When topiramate was discontinued there was an increase in the Negative Scale score (follow up score = 24). The dosage of topiramate was tailored cautiously by 25–50 mg every 4–7 days and the maximum dosage was 175 mg/day in two divided doses. No side effects were reported.

### Eating disorders and disordered eating

*McElroy et al. *[96] designed a randomized, placebo-controlled trial, investigating the therapeutic benefit of topiramate in treating binge eating disorder associated with obesity. For this 14-week, flexible dose (25–600 mg/day) trial, 61 outpatients (53 women and 8 men) with a body mass index of 30 or more, and a diagnosis of binge eating disorder according to the Structured Clinical Interview for DSM-IV were randomly assigned to receive topiramate (N = 30) or placebo (N = 31). The number of binges and binge days during the previous week were assessed at the initial screening visit together with psychiatric and medical history, physical examination, vital sign monitoring, routine blood chemical and haematological tests including fasting glucose, insulin and lipids, electrocardiogram and urinalysis. Monitoring of medication dose and compliance (review of patients' take-home diaries and tablet count), adverse events, use of non-study medications, weight and vital signs, efficacy measures, was achieved with regular visits. Topiramate was introduced at a dose of 25 mg/day and the dose titrated by 25 mg to 50 mg on day 4. It was then increased by 25–50 mg to 75–100 mg/day on day 7; the dose was subsequently increased by 50 mg/week for 4 weeks to maximum dose of 300 mg/day at 6 weeks and by 75 mg/week for 4 weeks to a maximum of 600 mg/day at 10 weeks. The dose was not changed from treatment period weeks 10 through 14 unless a medical reason supervened. If a patient did not tolerate any dose increase, the dose could be decreased to a tolerable one. The primary efficacy measure was binge frequency but the CGI severity scale, the Yale-Brown Obsessive Compulsive Scale (YBOCS) modified for binge eating, the Hamilton Depression Rating Scale, body mass index, weight were also used. Waist-to-hip ratio, percent and total body fat (measured by bioelectrical impedance), blood pressure, fasting blood glucose, insulin and lipids were also considered as secondary measures of efficacy at the last visit. Safety measures such as adverse events, clinical laboratory data, physical examination findings and vital signs were assessed. The baseline score on the YBOCS was 15 or more, suggestive of marked distress regarding binge-eating behaviour. Twenty-six subjects (42.6%) discontinued the study (Topiramate N = 14) but analysis included all patients with at least one post-randomization efficacy measure (intent to treat analysis) with a repeated-measures random regression with treatment-by-time as the effect measure. Topiramate was associated with a statistically significant reduction in binge eating frequency (topiramate 94% vs. placebo 46%) and binge day frequency (topiramate 93% vs. placebo 46%). The CGI severity scale and the Yale-Brown Obsessive Compulsive Scale showed improvement scores at the last visit and were greater in the treatment arm. The rate of decrease in Hamilton Depression Rating Scale scores did not differ between treatment groups. The mean weight loss for topiramate treated subjects was 5.9 kg compared to 1.2 kg in the placebo group. Median topiramate dose was 212 mg/day (range 50–600). Twenty-six patients discontinued. Nine patients (topiramate = 6) because adverse events with paraesthesias and headache as the most common side effects. Topiramate was associated with a significant change in diastolic blood pressure at the last visit compared with placebo among the intent to treat group. There was no significant difference between groups in mean change for the fasting metabolic measurements of insulin, glucose, LDL cholesterol, triglycerides and total cholesterol. *Hoopes et al.*, [97] enrolled 69 patients with DSM-IV bulimia nervosa in a randomised, double blind, placebo controlled trial. Sixty-four patients (33 in the placebo group vs. 31 in the topiramate group) were included in the intent to treat analysis. The primary efficacy measure, mean weekly number of binge and/or purge days, decreased 44.8% from baseline in the topiramate group versus 10.7% in the placebo group (p = 0.004). This was confirmed by significant reduction in scores on the Bulimic Intensity Scale, 37% for topiramate vs. 14% for placebo. The trial lasted for 10 weeks and the median dose was 100 mg/day (range 25–400). Topiramate, administered in monotherapy, was commenced at 25 mg/day for the first week. The dose was titrated by 25–50 mg increments per week to a maximum of 400 mg/day. Response supervened within 10 weeks. Only 3 patients discontinued from the trial (2 placebo, 1 topiramate) due to adverse events (topiramate: nausea). *Shapira et al. *[98] studied 13 female patients with binge eating disorder in a naturalistic, open label, add-on study. All the patients had co-morbid diagnoses. Treatment was begun at 25 mg/day and subsequently increased by 25–50 mg/week according to response and side effects to 1400 mg/day, given in divided doses. Response and side effects were valuated retrospectively as recalled by patients at monthly appointments. Outcome was measured as decrease in binge-eating episodes: none (0% to <25% reduction), mild (25 to <50% reduction), moderate (50 to <70% reduction), marked (75 to <100% reduction) or remission (complete cessation of binge eating episodes). Patient weight and BMI at beginning of treatment and at end point were recorded and statistically correlated. Nine patients displayed a moderate or marked response of binge eating disorder that was maintained for 18.7 +/- 8.0 months (range: 3 to 30 months), 7 continued to display the improvement at 21.1 +/- 6.0 (range 13–30 months), whilst 1 patient continued treatment because stabilised her bipolar disorder. Two patients displayed moderate or marked response that subsequently declined. The remaining two patients had a mild or no response. The mean topiramate dose was 492.3 +/- 467.8 mg/day for all 13 patients. The main weight at beginning of treatment was 99.3 +/- 26.4 kg and 87.5 +/- 20.4 kg at the end (z = -2.4, df = 1, p = .02) but only 7 patients lost 5 or more kg of weight. The mean dose of topiramate was higher in those who lost 5 kg or more (725.0 +/- 529.3 mg/day) compared to those who lost <5 kg (220.8 +/- 156.9 mg/day). Topiramate was well tolerated. However, 2 patients reported side effects (cognitive impairment and dyspepsia) which subsided with discontinuation and slower reintroduction of the dose. Two patients reported worsening of co-morbid bipolar (manic) symptoms. A mixed response of co-morbid condition was also noted (obsessive compulsive disorder, compulsive buying, major depressive disorder). *Barbee *[99] treated a series of five patients with adjunctive topiramate. All the patients had a long history of severe bulimia nervosa combined with significant different co-morbid conditions (major depression, bipolar disorder II, substance misuse, post traumatic stress disorder, dysthymia, social phobia, border line personality disorders and general anxiety disorder). The dose was titrated slowly to 95–400 mg/day according to clinical response. During a follow up period of 7–18 months, 3 patients responded to topiramate, 1 did not respond and 1 subject discontinued treatment because of gastro-intestinal related side effects. Only 1 case reported simultaneous improvement in the co-morbid affective disorder. Adverse events occurred in 2 patients (paraesthesias and constipation).

### Posttraumatic stress disorder

Berlant and van Kammen [100] retrospectively reviewed 35 patients with chronic posttraumatic stress disorder treated with topiramate as add-on treatment (N = 28) or monotherapy (N = 7). Dosage titration was slow with an initial dose of 12.5–25 mg/day, increased by 25–50 mg every 3–4 days until therapeutic response was achieved. The main duration of treatment was 33 weeks (range 1–119 weeks). Topiramate decreased nightmares in 79% (19/24) and flashbacks in 86% (30/35) of patients, with full suppression of nightmares in 50% and of intrusions in 54% of patients with these symptoms. Nightmares and intrusions partially improved in a median of 4 days (mean 11+/-13 days) and were fully absent in a median of 8 days (mean 35 +/- 49 days). Response was seen in 95% of partial responders at a dosage of 75 mg/day or less and in 91% of full responders at a dosage of 100 mg/day or less. The last 17 patients completed the PTSD Checklist-Civilian Version (PCL-C) before treatment and at week-4. Mean reduction in PCL-C score from baseline to week-4 was highly significant (baseline score = 60 vs. week-4 score = 39, p < .001), with similar reductions in re-experiencing, avoidance, and hyper-arousal criteria symptoms. Thirteen patients discontinued for various reasons during the study period. There were no serious side effects reported a part from a case of acute secondary narrow-angle glaucoma. Response assessment used the last observation carried forward.

### Alcohol dependence

Johnson and associated [101] conducted a double blind randomised controlled 12-week clinical trial comparing topiramate to placebo for treatment of 150 individuals with alcohol dependence. Of these 150 individuals, 75 were assigned to receive topiramate (escalating dose of 25–300 mg per day) and 75 had placebo as an adjunct to weekly-standardised medication compliance management. Primary variables were: self reported drinking (drinks per day, drinks per drinking day, percentage of heavy drinking day, percentage of day abstinent) and plasma gamma-glutamyl transferase as an objective index of alcohol consumption. The secondary efficacy variable was self-reported craving measured on the 14-item obsessive compulsive drinking scale. In the topiramate group 55 subjects completed the study versus 47 in the placebo group. The authors adopted intention to treat analysis. Response supervened between 6 and 8 weeks. At study end, participants on topiramate, compared with those on placebo, had 2.88 (95% CI -4.50 to -1.27) fewer drinks per day (p = 0.0006), 3.10 (-4.88 to -1.31) fewer drinks per drinking day (p = 0.0009), 27.6% fewer heavy drinking days (p = 0.0003), 26.2% more days abstinent (p = 0.0003), and a log plasma gamma-glutamyl transferase ratio of 0.07 (-0.11 to -0.02) less (p = 0.0046). Topiramate induced differences in craving were also significantly greater than those of placebo, of similar magnitude to the self-reported drinking changes, and highly correlated with them. There were no discontinuations due to side effects and topiramate was generally well tolerated.

### Gilles de la Tourette's syndrome

Abuzzahab et al. [102] described 2 cases of Tourette's syndrome successfully treated with topiramate respectively at 50–200 mg for 8 months and 100 mg nocte for a month. In both cases, previous medication were tapered down and discontinued during the first two weeks of treatment. Significant weight loss was noted: weight dropped from 183 to 145 lb for case 1 and 12.5 lb weight loss for case 2. Lacks of concentration, loss of appetite, thirst and lethargy sensitive to dose reduction were reported.

### Emotional unstable personality disorder

*Cassano et al. *[103] described a case of bipolar mood disorder and border line personality disorder complicated by self mutilating behaviour, which responded to topiramate administration with an on-off-on design. Although depressive symptoms persisted, topiramate controlled self-injurious acts within 2 weeks at a dose of 200 mg/day. No side effects were reported. Teter et al. [104] published a case of an inpatient with psychotic disorder not otherwise specified and border line personality disorder treated with topiramate at the dose of 200 mg/day. Borderline symptoms improved in 6 weeks. Considerable weight loss was also reported.

### Adverse events, safety and tolerability

Topiramate was generally well tolerated. General observations suggested that side effects occurred with high dose titration and frequently resolved or lessened with time and/or dosage reduction. Conversely, slow dose titration was associated with a lower rate of side effects [e.g. [73,75,78,84]. This is in agreement with data from epilepsy clinical trials, which suggest possible appearance of adverse reactions and treatment discontinuation following rapid dose titration and a target dose greater than 400 mg/day [105-109]. This indicates that individuals on complex pharmacological treatments are more vulnerable to side effects, particularly with sodium valproate and lithium [80]. The commonest adverse events (table 1) across the studies analysed in this review were paraesthesias/numbness (N = 116, 12.9%), nausea and vomiting (N = 56, 6.2%), cognitive impairment (N = 48, 5.4%), headache (N = 46, 5.1%), dizziness (N = 45, 5.0%), sedation/drowsiness (N = 44, 4.9%), fatigue (N = 38, (4.2%), decreased appetite (N = 24, 2.7%), frequent peristalsis (N = 20, 2.2%), somnolence (N = 19, 2.1%), blurred vision (N = 18, 2.0%), slow memory (N = 16, 1.8%). There was one reported case of psychotic features [71], a case of delirium in a patient who overmedicated with 800 mg of topiramate and tranylcypromine sulphate (170 mg) combined with alcohol [76], a case of acute narrow angle glaucoma [100] and 2 cases of hematuria [92]. Occurrence of hematuria is consistent with the known 2 to 4 increased risk of nephrolithiasis during topiramate treatment [45]. Rare but serious adverse events have been described with topiramate (e.g. metabolic acidosis, acute myopia, acute glaucoma, oligohidrosis, hyperthermia) leading topiramate to be under review by regulatory authorities in several jurisdictions.

**Table 1:** Adverse events in order of frequency

Adverse events *	**Topiramate (N = 896) *N (%)***
Paresthesia/numbness	116 (12.9)
Nausea/vomiting	56 (6.2)
Cognitive impairment	48 (5.4)
Headache	46 (5.1)
Dizziness	45(5.0)
Sedation/drowsiness	44 (4.9)
Fatigue	38 (4.2)
Decreased appetite	24 (2.7)
Frequent peristalsis	20 (2.2)
Somnolence	19 (2.1)
Blurred vision	18 (2.0)
Slow memory	16 (1.8)
Lack of concentration	11 (1.2)
Influenza-like-symptoms	10 (1.1)
Panic/anxiety	9 (1.0)
Dysgeusia	8 (0.9)
Dry mouth	8
Nervousness	7 (0.8)
Rash	7
Ataxia	7
Insomnia	7
Constipation	7
Reduced libido	5 (0.6)
Memory concerns	5
Dyspepsia	5
Unwanted weight loss	4 (0.4)
Increased thirst	4
Word-finding difficulty	4
Impaired concentration	4
Tremor	4
Itching	4
Sweating	3 (0.3)
Confusion	3
Slowed thinking	3
Psychosis	3
Slurred speech	3
Increased salivation	3
Sleep disturbance	3
Backache	3
Increased appetite	2 (0.2)
Gastrointestinal disturbances	2
Agitation	2
Cold sensitivity	2
Worsening of symptoms	2
Increased libido	2
Amenorrhea	1 (0.1)
Hematuria	1
Dysuria	1
Urticaria	1
Increased suicidality	1
Glaucoma	1
Water retention	1
Delirium	1
Grand mal seizures	1

(*) From the studies reviewed only

### Weight loss

Topiramate weight loss was reported in 21 of the 32 studies analysed [70,72,75,78-84,86-89,91,92,96,98,101,102,104] and reached 5% reduction of the baseline weight prior to treatment initiation in 5 studies [75,79-81,92]. Weight change was not systematically evaluated in 11 trials [71,76,85,90,93-95,99,97,100,103]. Among the studies, a frequent finding was that the initial BMI affected topiramate-induced weight loss and that greater weight loss was associated with higher BMI at baseline [e.g. [73,78,80,81,86]. In diabetic patients, topiramate induced weight loss was also associated with glycemic control and normalization of blood pressure in hypertensive subjects [78,96].

## Conclusion

Preliminary reports [70-72], available for review, suggested a trend towards improvement in acute mania but more recent unpublished controlled studies, not available for review, showed lack of efficacy [[74]; Calabrese, personal communication]. It emerges that in the light of current evidence, there is limited scope for the use of topiramate in acute mania. The only randomised single blind study by McIntyre et al. [88], in the treatment of refractory bipolar disorders in depressive phase, showed a significant improvement in 56% of the subject treated with topiramate versus 59% in the bupropion group. This study, according to the authors, was not powered to detect a difference in efficacy between the two treatment groups and, given the small sample size, it only aimed to corroborate the antidepressant property of topiramate already shown in naturalistic studies [89]. If demonstrated efficacious in further adequately powered controlled studies, topiramate could fill the therapeutic vacuum in the treatment of bipolar depression as alternative or adjuvant to mood stabilisers. The role of topiramate in the treatment of rapid cycling bipolar disorders [75], and as adjunctive treatment in refractory bipolar disorder in adults [76-87] and children [90], is limited by the open label nature of the published studies: lack of randomisation and blindness, heterogeneous patient, population resistant to conventional treatment regimes, incomplete information on current or past treatment for illness, concomitant medications with possibly inflating side effects profile and therapeutic effect, self-reported weight and side effects, qualitative assessment of response to treatment, various settings and variegated level of symptoms, co-morbid psychiatric and medical conditions. Although there is no sufficient evidence for its use in these conditions, its trend towards improvement warrants controlled studies. However, it may not be sustained in randomised studies as observed in acute mania. The effectiveness of topiramate in schizophrenia is similarly based on uncontrolled studies. Only Drapalski et al. [95] reported a positive outcome in treatment of negative symptoms with adjunctive topiramate. Millson et al. [93] observed a post-treatment deterioration in PANSS scores in 5 patients treated with topiramate. Dursum and Deakin [94] also noted no reduction in BRPS scores when topiramate was augmented with antipsychotic medications. These controversial results, conveys doubts about the efficacy of topiramate in schizophrenia and uncertain the postulated stabilizing properties of topiramate in the interaction between the glutamatergic and dopaminergic systems. Alternatively, these observations may reflect that patients analysed were a heterogeneous group in many aspects of their illness and future studies would probably require more strict research criteria. Evidence for the use of topiramate in binge eating disorders and bulimia nervosa is encouraging and suggest a complementary role of topiramate in the treatment of these conditions together with established treatment strategies (e.g. SSRIs). McElroy et al. [96], reported efficacy of topiramate in reducing binge eating episodes by 93% in the treatment arm compared to 46% in the placebo group. Hoopes at al. [97] reported a decrease in the mean weekly number of binge and/or purge days by 44.8% from baseline in the topiramate group versus 10.7% in the placebo group (p = 0.004) and a significant reduction in scores on the BIS by 37% in the topiramate group vs. 14% in the placebo group. The only study in the treatment of PTSD by Berlant and van Kammen [100] was suggestive of efficacy in treating nightmares (79%) with full suppression in 50% of cases, flashbacks (86%) and intrusions (54%). The relative short duration of the trial did not allow exploration of a possible prophylactic role of topiramate. However, similarly to bipolar spectrum disorder, the naturalistic nature of this report constitutes a limitation for its validity. The only study by Johnson et al. [101] in the treatment of alcohol dependence was controlled. It indicated that topiramate, as an adjunct to standardised medication, is more efficacious in reducing alcohol consumption than placebo. This study warrants further investigation and indicates that topiramate could be included in the rather limited pharmacological armamentarium to defeat alcohol dependence. The effectiveness of topiramate in unipolar depression [91,92], emotionally unstable personality disorder [103,104] and Gilles de la Tourette's syndrome [102] is entirely based on case reports and case series. The evidence is sometimes controversial and at the time of writing there is no clear indication for treatment with topiramate. Weight change was not always systematically reported across the studies. However, findings are encouraging considering the rather disappointing success rates of efficacious prevention programs [110] and have potential implications in reversing increased body weight and obesity induced by psychotropic medication [111,112]. Weight loss was also proportional to the initial BMI and it was associated with glycemic control and normalization of blood pressure in hypertensive subjects. Topiramate was generally well tolerated and serious adverse events were rare. Polypharmacy often contributed to an increased rate of side effects.

## Competing interest

The author(s) declare that they have no competing interests.

## Drug names

Topiramate (Topamax)

## Supplementary Material

Additional File 1 Details of published studies included in the review. Click here for file
