# Supplementary material for: Review of the use of Topiramate for treatment of psychiatric disorders
Source: Ann Gen Psychiatry. 2005 Feb 16;4:5. doi: 10.1186/1744-859X-4-5 (PMC1088011; doi:10.1186/1744-859X-4-5)
Supplement: Additional File 1 — Details of published studies included in the review. [file 1744-859X-4-5-S1.doc]

# Table 3

**Bipolar disorders**

| **Study**  Characteristics | **Design**  Type, duration, dose (range) | **Outcome**  Response rate % (N) | **Comments** | **Weight loss** |
| --- | --- | --- | --- | --- |
| **Calabrese (70)**  **(2001)**  Total Number: 11  Discontinued: 6  Settings: inpatients  Diagnosis: BP I  Rating scales: YMRS, HAM-D-17, BPRS, GAS, CGI-I | Open label  Monotherapy  Duration: 28 days  Dose: 313 mg/day (50-612 mg/day) | 60% (N=3) response rate | Mild symptoms  Response in 4 weeks  Well tolerated | Weight loss reported |
| **Grunze (71)**  **(2001)**  Total Number: 11  Discontinued: 2  Settings: inpatients  Diagnosis: BP (mania)  Rating scales: YMRS, CGI-BD, HAM-D-21 | Open label  Adjunctive treatment  on-off-on design  Duration: 4 weeks  Dose: 200 mg/day  (50-200 mg/day) | 89% response rate (N=8) following reintroduction of topiramate | Refractory patients  Response in 1 week  Well tolerated | Not evaluated |
| **Bozikas (72)**  **(2002)**  Total Number: 14  Discontinued: 1  Settings: inpatients  Diagnosis: BP (manic)  Rating scales: BRMAS | Open label  Monotherapy (N=9)  Adjunctive or co-administered (N=5)  Duration: 4 weeks  Dose: 310 mg/day  (150-700 mg/day) | Response rate was 61.5% (N=8) | Well tolerated  Benzodiazepines allowed in the study  Response in 1-2 weeks | Weight loss reported. BMI >30 in patients who lost weight. |
| **Kusumakar (75)**  **(1999)**  Total Number: 27 (Women)  Discontinued: 4  Settings: outpatients  Diagnosis: BP I/II, rapid cycling  Rating scales: YMRS, HAM-D | Open label  Adjunctive treatment  Duration: 16 weeks  Dose: 100-150 mg/day | 65% (N=15) achieved euthymia | Response within 12 weeks  N=4 discontinued for adverse events  Refractory patients | Weight loss reported; More than 5% in 9 subjects. |
| **Marcotte (76)**  **(1998)**  Total Number: 58  Discontinued: 6  Settings: in/outpatients  BP-RC, NOS, SZA, dementia  Rating scale: global qualitative assessment including a Likert scale | Retrospective  Adjunctive treatment  Duration: 16 weeks  Dose: 200 mg/day (25-400) | Marked to moderate improvement in 62% (N=36), 52% in the BP-RC sub-population | Response within 72 hrs  N=6 discontinued for adverse events  Refractory patients | Not evaluated |
| **Chengappa (78)**  **(1999)**  Total Number: 20  Discontinued: 0  Settings: in/outpatients  Diagnosis: BP I, SZA-BP  Rating Scales: YMRS, CGI-BP, HAM-D | Open label  Adjunctive treatment  Duration: 142 days (29-293)  Dose: 210.5 mg/day (50-300 mg/day) | 60% (N=12) response rate | Response in 2-4 weeks  Well tolerated  Refractory patients | Weight loss reported. Greater weight loss with BMI of 30 or greater. |
| **McElroy (79)**  **(2000)**  Total Number: 56  Discontinued: 29  Settings: outpatients  Diagnosis: BP and SZA-BP  Rating scales: YMRS, CGI-BP, IDS | Open label  Adjunctive treatment  Duration: 294.6 days (SD=145.3)  Dose: 244.7 mg/day (SD=241.7) | 40.5% response rate with 63.3% (N=19) manic patients, and 27.3% (N=3) depressed responded at 10 weeks | Mild symptoms  Refractory patients  Response in 4-10 weeks  N=6 discontinued for adverse events | 5% change in BMI at last valuation (N=52) |
| **Sachs (80)**  **(2002)**  Total Number: 14  Discontinued: 7  Settings: not specified  Diagnosis: BP I/II  Rating scales: CGI, GAF, SCID | Chart review  Adjunctive therapy  Duration: 22.4 weeks (SD=22.0)  Dose: 50 mg/day (SD=27.4) | 36% response rate (N=4) | Refractory patients  Response in >2 weeks  N=5 discontinued for adverse events | 5% or greater change in weight (N=4, BMI ≥28) |
| **Eads (81)**  **(2000)**  Total Number: 17  Discontinued: 8  Settings: Not specified  Diagnosis: BP I/II  Rating Scales: GAF, CGI-I | Open label  Retrospective  Adjunctive treatment  Duration: 22.4 weeks (SD=22.0)  Dose: 826 mg/day | 53% (N=9) response rate. | Refractory patients  N=8 discontinued for adverse events | 5% or greater change in weight (N=4, BMI ≥ 28) |
| **Ghaemi (82)**  **(2001)**  Total Number: 76  Discontinued: 39  Settings: outpatients  Diagnosis: BP I/II or NOS  Rating scales: CGI-I | Chart review  Monotherapy (N=6) or adjunctive therapy (N=70)  Duration: 17.5 weeks (SD=16.7)  Dose: 96.1 mg/day (SD=94.19) | 13.2% (N=10) response rate associate to higher doses | Refractory patients  Response in 2 weeks  N=27 discontinued for adverse events | Weight loss reported and associated with higher doses |
| **Vieta (83)**  **(2001)**  Total Number: 21  Discontinued: 6  Settings: Not specified  Diagnosis: BP I/II, SZA-BP  Rating scales: YMRS, CGI, HDRS-17 | Open label  Adjunctive treatment  Duration: 6 weeks  Dose: 158.33 mg/day (SD=45.6) | 28.5% (N=6) response rate | Refractory patients  Response in 1-2 weeks  N=1 discontinued for adverse events | Weight loss reported |
| **Saxena (84)**  **(2002)**  Total Number: 9  Discontinued: 1  Settings: not specified  Diagnosis: BP  Rating scales: CGI-I, GGS, YMRS, HAM-D | Open label  Adjunctive treatment  Duration: 10-24 weeks  Dose: 488 mg/day  Baseline assessment not available | 44% (N=4) response rate | Well tolerated  Response in 4-8 weeks  N=1 discontinued for adverse events | Weight loss reported. |
| **Vieta, Torrent (85)**  **(2002)**  Total Number: 34  Discontinued: 8  Settings: not specified  Diagnosis: BP II  Rating scales: YMRS, HDRS, CGI-BP | Open label  Adjunctive treatment  Duration: 24 weeks  Dose: 202 mg/day (SD=65) | Fifty-nine percent of manic patients and 55% of depressed patients responded | Refractory patients  LOCF method  Response within 2-6 weeks  N=1 discontinued for side effects | Not specified |
| **Vieta, Ros (86)**  **(2002)**  Total Number: 61  Discontinued: 6  Settings: multicentre study  Diagnosis: BP II  Rating scales: YMRS, HDRS, CGI-BP | Open label  Adjunctive  Duration: 12 weeks  Dose: 214 mg/day | 70% (N=43) response rate | Refractory patients  LOCF method  Response in 12 weeks  N=1 discontinued for adverse events | Weight loss reported and greater with higher BMI at baseline (>40) |
| **McIntyre (87)**  **(2002)**  Total Number: 109  Discontinued: 10  Settings: not specified  Diagnosis: BP II  Rating scales: YMRS, MADRAS, CGS | Open label  Adjunctive treatment  Duration: 16 weeks  Dose: 140.8 mg/day (25-400 mg/day) | 70% (N=69) response rate | Refractory patients  Response in 2-16 weeks  N=12 discontinued for adverse events | Weight loss reported. |
| **McIntyre (88)**  **(2002)**  Total Number: 36 (topiramate N=18)  Discontinued: 13 (topiramate N=8)  Settings: outpatients  Diagnosis: BP I/II (depression)  HDRS-17, YMRS, CGI-I | Randomised single-blind (topiramate v. bupropion SR)  Add on design  Duration: 8 weeks  Dose: 176 mg/day (SD=102) | Response rate was 56% (N=10) for topiramate and 59% (N=13) for bupropion | Mild symptoms  Response in 2-4 weeks    N=6 discontinued for adverse events | Weight loss reported |
| **Hussain (89)**  **(2001)**  Total Number: 83  Discontinued: 42  Settings: not evaluated  Diagnosis: BP I/II (depressive phase)  Rating scales: HAM-D-17 | Open label  Adjunctive treatment  Duration: 3 years  Dose: 275 mg/day (Range 100-400) | 85% (N=35) response rate | Refractory patients  Response in 4 weeks  N=19 discontinued for adverse events | Weight loss reported |
| **DelBello (90)**  **(2002)**  Total Number: 26  Discontinued: 0  Settings: outpatients  Diagnosis: children and adolescent with BP  Rating scales: CGI, CGS | Chart Review  Adjunctive treatment  Duration: 4.1 months (SD=6.1)  Dose: 104 mg/day (SD=77) | 62% response rate for overall illness and 73% for mania | Well tolerated  Response onset not specified | Not evaluated |

Unipolar depression

| **Study**  Characteristics | **Design**  Type, duration, dose (range) | **Outcome**  Response rate % (N) | **Comments** | **Weight loss** |
| --- | --- | --- | --- | --- |
| **Gordon (91)**  **(1999)**  Total Number: 1  Settings: not specified  Diagnosis: MD (in remission) and obesity  Rating scales: none | Case series  Adjunctive treatment  Duration: 8 weeks  Dose: 300 mg/day | Worsening of anxiety and depressive symptoms | Response in 2-3 months  Concurrent medication change | Weight loss reported |
| **Carpenter (92)**  **(2002)**  Total number: 16 (women)  Settings: not specified  Diagnosis: MD and obesity  Rating scales: none | Chart review  Adjunctive treatment  Duration: 17.7 weeks (SD=13.4)  Dose: 277±101 mg/day (100-400) | 44% (N=7) responded at end point | Refractory patients  Response in 5.5 weeks  N=4 discontinued for adverse events | Weight loss reported >5% |

Schizophrenia

| **Study**  Characteristics | **Design**  Type, duration, dose (range) | **Outcome**  Response rate % (N) | **Comments** | **Weight loss** |
| --- | --- | --- | --- | --- |
| **Millson (93)**  **(2002)**  Total Number: 5  Settings: inpatients  Diagnosis: chronic SCH  Rating scales: PANSS | Case series  adjunctive treatment  Duration: 1 month  Dose: 250 mg/day  (200-300 mg/day) | Deterioration in condition in all the 5 patients | Refractory patients  Well tolerated | Not evaluated |
| **Dursun (94)**  **(2001)**  Total Number: 26  (9 on topiramate)  Discontinued: none  Settings: outpatients  Diagnosis: SCH  Rating scales: BPRS | Case series  Adjunctive treatment with either topiramate or lamotrigine Duration: 24 weeks  Dose: 225-300 mg/day | No significant reduction in symptoms | Refractory patients  Well tolerated | Not evaluated |
| **Drapalski (95)**  **(2001)**  Total Number: 1  Settings: outpatient  Diagnosis: chronic SCH  Rating scales: PANSS | Case report  Adjunctive treatment  Dose: 175 mg/day  Duration: 17 weeks | Dramatic improvement of negative symptoms | Refractory patient  Well tolerated  Response in 4-8 weeks | Not evaluated |

**Eating disorders and disordered eating**

| **Study**  Characteristics | **Design**  Type, duration, dose | **Outcome**  Response rate % (N) | **Comments** | **Weight loss** |
| --- | --- | --- | --- | --- |
| **McElroy (96)**  **(2003)**  Total Number: 30 (topiramate) and 31 (placebo)  Discontinued: 26  Settings: outpatients  Diagnosis: BED and obesity  Rating scales: CGI, HDRS, YBOCS | Randomized, placebo controlled  Duration: 14 week  Dose: 25-600 mg/day (median dose 212 mg/day) | Reduction in binge frequency in the topiramate group (N=28) | Response in 1-6 weeks  Intent to treat analysis  N=14 (topiramate arm) discontinued | Weight loss reported |
| **Hoopes (97)**  **(2003)**  Total Number: 35 (topiramate) and 34 (placebo)  Discontinued: 3  Settings: outpatients  Diagnosis: BN  Rating scales: BIS | Randomised, placebo controlled  Duration: 10 week  Dose: 25-400 mg/day (median dose 100 mg/day) | Reduction in BIS in the topiramate group (37%) versus the control group (14%) | Intent to treat analysis  Well tolerated  Response in 10 weeks | Not evaluated |
| **Shapira (98)**  **(2000)**  Total Number: 13 (female)  Discontinued: 0  Settings: outpatients  Diagnosis: BED  Rating scales: none | Open label  Adjunctive treatment  Duration: 21.1+/-6.0 months  Dose: 492.3 mg/day  (SD=467.8 mg/day)  (Range 100-1400 mg/day | Improvement in 54% (N=7) | Response within 3 months  Well tolerated | Weight loss reported. More weight loss with higher dosage. |
| **Barbee (99)**  **(2003)**  Total number: 5  Discontinued: 0  Settings: not specified  Diagnosis: BN with MD, BP II, dysthymia, substance misuse, social phobia, BLPD, GAD, PTSD  Rating scales: none | Case series  Adjunctive treatment  Duration: 7-18 months  Dose: 200 mg (case 1,2), 90 mg/day (case 3), 400 mg/day (case 4) | Improvement of binging and purging in three cases (cases 1,2,4) with some improvement in co-morbid affective disorders in case 4 | Refractory patients  Adverse events reported (case 3 and 5)  Response in 4 weeks | Not reported |

Posttraumatic stress disorder

| **Study**  Characteristics | **Design**  Type, duration, dose (range) | **Outcome**  Response rate % (N) | **Comments** | **Weight loss** |
| --- | --- | --- | --- | --- |
| **Berlant (100)**  **(2002)**  Total Number: 35  Discontinued: 13  Settings: not specified  Diagnosis: chronic PTSD  Rating scales: PCL-C | Open label  Adjunctive treatment or  Monotherapy  Duration: 33 weeks  (1-119)  Dose: 12.5–500 mg/day | Improvement of flashbacks in 86% (30/35) of patients and nightmares in 79% (19/24) of patients | Refractory patients  LOCF method  Response in 1-3 weeks  N=9 discontinued for adverse events | Not reported |

Alcohol dependence

| **Study**  Characteristics | **Design**  Type, duration, dose (range) | **Outcome**  Response rate % (N) | **Comments** | **Weight loss** |
| --- | --- | --- | --- | --- |
| **Johnson (101)**  **(2003)**  Total Number: 75 topiramate, 75 placebo  Discontinued: 47 (topiramate N=20)  Settings: out patients  Diagnosis: AD  Rating scales: OCDS | Randomised, double blind, placebo controlled  Monotherapy  Duration: 12 weeks  Dose: 300 mg/day | Significant reduction in primary outcome measures in the topiramate group | Brief behavioural treatment also administered  Response in 6-8 weeks  LOCF analysis  Well tolerated | Weight loss reported |

Gilles de la Tourette’s syndrome

| **Study**  Characteristics | **Design**  Type, duration, dose (range) | **Outcome**  Response rate % (N) | **Comments** | **Weight loss** |
| --- | --- | --- | --- | --- |
| **Abuzzahab (102)**  **(2001)**  Total Number: 2  Settings: not specified  Diagnosis: GTS  Rating scales: none | Case series  Monotherapy  Duration: 32 weeks and 4 weeks  Dose; 50-200 mg/day and 200-100 mg/day | Control of symptoms | Response in 1-4 weeks  Well tolerated | Weight loss reported |

Emotionally unstable personality disorder

| **Study**  Characteristics | **Design**  Type, duration, dose (range) | **Outcome**  Response rate % (N) | **Comments** | **Weight loss** |
| --- | --- | --- | --- | --- |
| **Cassano (103)**  **(2001)**  Total Number: 1  Settings: not specified  Diagnosis: BP II (MD), BLPD  Rating scales: none | Case report, on-off-on design  Duration: not specified  Dose: 200 mg/day | Improvement of self injurious behaviour but not symptoms of depression | Well tolerated  Response in 2 weeks | Not reported |
| **Teter (104)**  **(2000)**  Total Number: 1  Settings: inpatient  Diagnosis: NOS, BL PD, obesity  Rating scales: none | Case report  Adjunctive treatment  Duration: 10 weeks  Dose: 200 mg/day  (50-325 mg/day) | Improvement of symptoms | Response in 6 weeks  Well tolerated | Weight loss reported |

**BP I or II:** bipolar affective disorder type I or II, **BP-RC:** rapid cycling bipolar mood disorder, **SZA:** schizoaffective disorder, **SZA-BP:** schizoaffective disorder, bipolar type, **SCH:** schizophrenia, **MD:** major depression, **AD:** alcohol dependency, **GTS:** Gilles de la Tourette’s syndrome, **BED:** binge-eating disorder, **BN:** bulimia nervosa, **NOS:** psychotic disorder not otherwise specified, **BLPD:** border line personality disorder, **PTSD:** post traumatic stress disorder, **IDS:** Inventory of depressive symptoms, **YMRS:** young mania rating scale, **BRMAS:** Bech-Rafaelsen Mania Assessment Scale, **MADRAS:** Montgomery and Asberg depression rating scale, **BPRS:** brief psychiatric rating scale, **GAS:** global assessment scale, **GAF:** Global assessment of functioning scale**, CGI-BP:** Clinical global impression scale modified for bipolar mood disorder, **CGI-I:** Clinical global impression scale for improvement, **HAM-17 or 21:** 17 or 21 item Hamilton depression rating scale, **PANSS:** Positive and negative syndrome scale, **SCID:** Structured clinical interview for DSM IV-R, **YBOCS:** Yale Brown Obsessive Compulsive Scale (modified for binge eating disorders), **OCDS:** obsessive compulsive drinking scale, **LOCF method:** last observation carried forward, **BIS:** bulimic intensity scale, **PCL-C:** PTSD Checklist-Civilian Version.

Additional file 1

Table 3 – Details of published studies included in the review.

File type: DOC
